# Transformer-Based Network with Optimization for Cross-Subject Motor Imagery Identification

**DOI:** 10.3390/bioengineering10050609

**Published:** 2023-05-18

**Authors:** Xiyue Tan, Dan Wang, Jiaming Chen, Meng Xu

**Affiliations:** Faculty of Information Technology, Beijing University of Technology, Beijing 100124, China

**Keywords:** brain computer interface (BCI), EEG signal, transformer, self-attention, motor imagery (MI)

## Abstract

Exploring the effective signal features of electroencephalogram (EEG) signals is an important issue in the research of brain–computer interface (BCI), and the results can reveal the motor intentions that trigger electrical changes in the brain, which has broad research prospects for feature extraction from EEG data. In contrast to previous EEG decoding methods that are based solely on a convolutional neural network, the traditional convolutional classification algorithm is optimized by combining a transformer mechanism with a constructed end-to-end EEG signal decoding algorithm based on swarm intelligence theory and virtual adversarial training. The use of a self-attention mechanism is studied to expand the receptive field of EEG signals to global dependence and train the neural network by optimizing the global parameters in the model. The proposed model is evaluated on a real-world public dataset and achieves the highest average accuracy of 63.56% in cross-subject experiments, which is significantly higher than that found for recently published algorithms. Additionally, good performance is achieved in decoding motor intentions. The experimental results show that the proposed classification framework promotes the global connection and optimization of EEG signals, which can be further applied to other BCI tasks.

## 1. Introduction

Brain-computer interface (BCI) is a system that directly interacts with the outside world without relying on peripheral nerves and muscles to output information, creating new hands-free interaction paradigms [1]. Researchers obtain the user’s intentions by extracting brain signals to control the BCI system, and design meaningful artificial intelligence experiences [2]. Examples include wheelchairs controlled using EEG signals, brain-controlled prosthetics, robotic arms, robots, and Augmented Reality [3,4]. BCI systems that rely on EEG control are used as auxiliary tools to help paralyzed or stroke patients during rehabilitation, thereby improving their quality of life [5,6,7]. Therefore, BCI research has become a hot topic in rehabilitation medicine.

Motor imagery (MI) EEG signals generated in the motor cortex are one of the most widely studied BCI paradigms [8]. The power of alpha (8–13 Hz) and beta (14–30 Hz) rhythms is inhibited or promoted in the sensorimotor cortex of the brain and the amplitude of the EEG signal decreases or increases, accompanied by the phenomenon of spectral oscillation when the user imagines or performs an action with their hands, feet, or tongue [9]. MI mainly induce event-related desynchronization (ERD)/Event-related synchronization (ERS) in the alpha and beta bands. The body part of the subject that wants to produce movement can be identified by accurately classifying EEG signals related to MI. MI-BCI has been widely used and achieved good results in smart healthcare applications, such as post-stroke rehabilitation and mobile assistive robots [10]. However, the complex brain neural electric field activity results in the relatively poor performance of existing computer-aided classification frameworks.

Methods based on traditional machine learning have been widely used in the feature extraction and classification of EEG signals. The common spatial pattern (CSP) [11] and other variant methods, such as the filter bank common spatial pattern (FBCSP) [12], are mostly applied to the spatial distribution extraction of multi-channel EEG data features. The main concept of the CSP method is to create an optimal public space filter with category information under supervision. The eigenvectors and maximum of the different variance values of two types of signals are obtained as the input of the classifier by simultaneously diagonalizing the covariance matrix of the two types of tasks. CSP mainly uses the spatial distribution difference of energy, which is straightforward and effective but ignores the time–frequency characteristics of the signal. The FBCSP algorithm is based on a common spatial pattern and was proposed to filter EEG signals with nine groups of band-pass filters of different frequency bands, extract the nine respective CSP mode algorithms features, select features in combination with mutual information, and classify the extracted feature vectors into two categories [13]. A sub-band common spatial pattern (SBCSP) decomposes EEG signals into sub-bands using a filter bank, applies discriminant analysis to extract features, and feeds these features into linear discriminant analyzers (LDA) to obtain scores that are fused to obtain a decision [14]. Jin et al. used the Pearson correlation coefficient to select the channel containing the most relevant information and used a regularized common spatial pattern (RCSP) to extract effective features. In addition, a support vector machine (SVM) was used as the classifier [15]. However, the sample size of MI datasets has typically been small, and the feature selection of this type of method relies heavily on artificial markers that easily overfit small datasets, leading to unsatisfactory classification accuracy [16].

In recent years, deep learning methods have demonstrated excellent performance in various medical applications [17]. Researchers have found that deep learning methods perform better in MI classification tasks than traditional machine learning methods [18,19]. CNNs can effectively perceive the features of a local domain and obtain deeper feature representations. In the BCI, researchers have used CNN to establish an end-to-end EEG decoding model, and deep learning (DL) has been used for automatic feature extraction [20] and classification [21,22], achieving leading performance [23]. Schirrmeister et al. proposed the DeepConvNet and ShallowConvNet algorithms, which stack the temporal sinc-convolutional layer and spatial depthwise convolutional layer to decode the characteristics of motor imagery EEG signals [24]. Sakhavi et al. proposed channel-wise convolution with a channel mixing method combining FBCSP and CNN that extracts features using the FBCSP algorithm. The signal envelope is extracted using the Hilbert transform, utilizing the neural network as a classification model. This approach achieved reasonable accuracy [25]. Lawhern et al. proposed the EEGNet algorithm, which combines the idea of FBCSP and a lightweight neural network. The size of the model is much smaller than DeepConvNet and ShallowConvNet, and the model applied to motion imagery and other BCI paradigms achieved fine classification accuracy [22]. Employing deep CNN for EEG classification is a promising classification technology that is superior to traditional machine learning methods [26]. In the convolution process, the internal relations of signals can be sensed by increasing the number of convolution layers. However, some details are lost with a gradual increase in the receptive field. Additionally, a deeper network structure leads to a large number of calculations, which increases the complexity and running time [27]. A recurrent neural network (RNN) is utilized to solve this problem and learn the temporal characteristics of EEG signals, which can mine temporal information from data [28]. Sun et al. proposed the CostNet method, a novel RNN-based network with horizontal and vertical cross-connections that effectively extract spatial and temporal representations to reduce the gradient propagation difficulty [29]. However, RNN has the problems of gradient disappearance and gradient explosion and lacks the ability to process long time series data. Therefore, researchers have used the long short-term memory (LSTM) method to control information state by gate [30]. Wang et al. used one-dimension aggregate approximation to extract the features of EEG signals. Subsequently, the long-term dependence and classification were learned using LSTM [31]. Tsiouris et al. extracted features of EEG signals in the time and frequency domains, obtained functional connection strength through the graph theory, and used two-layer LSTM to predict epilepsy [32]. A gated recurrent unit (GRU) is a variant of the LSTM method that has a faster convergence rate while reducing the number of LSTM parameters [33]. The study [34] proposed a deep learning classifier based on CNN and LSTM to detect MI-EEG for left and right hands, which is a promising discovery. Luo et al. tested LSTM and GRU models by classifying the features extracted by FBCSP, indicating that the GRU model achieved better results in EEG classification [35]. However, these methods are only suitable for small datasets, and their efficiency is unsatisfactory because the RNN procedures cannot be parallelized. In addition, the output stage of RNN only integrates the current time point and its previous information. CNN and RNN have limitations in sensing the overall dependence of EEG signals, which may lead to performance degradation of the EEG recognition framework.

Recently, self-attention mechanism has been applied to machine translation with satisfactory results. The attention mechanism of deep learning was inspired by attention in biology. Through the attention mechanism, the model can dynamically assign a weight to the input vector and assign a higher weight to the part that contributes most to classification or prediction to improve the classification accuracy and expand the interpretability of the model [36]. Transformer model [37] was proposed in 2018, which abandoned the structure of RNN and CNN and directly used the attention mechanism. This study first proposes the use of self-attention mechanism to extract global dependence of input, which has the following advantages. First, the interpretability of the model is expanded. Compared with RNN, the information stored by the hidden layer is not easy to visualize, and the attention distribution of the model on the data can be analyzed by visualizing the correlation weight. Second, its single-layer computational complexity is less than that of an RNN, and its structure can be used for parallel computing. Third, the correlation between any two units can be calculated, which can better solve the long-term dependence problem. However, its ability to extract local details is relatively weak because the transformer attention structure completely abandons the structure of convolutional network. Transformer model has rarely been studied in the field of BCI, and the attention mechanism may be helpful for decoding EEG signals more reasonably.

To overcome these above limitations, we propose a VAT-TransEEGNet algorithm to decode EEG signals, employs a VAT method to regularize constraints, and uses a Particle Swarm optimization (PSO) for optimization. Specifically, the algorithm first filters raw EEG signals following the idea of Butterworth band-pass filter. Next, a depthwise convolution attention block is carried out along the temporal dimension, attention score of each channel is calculated for the feature extraction and aggregation. Subsequently, a separable convolution attention block is carried out along the spatial dimension, the useful representation for classification is obtained by calculating the attention intensity between different time points. Finally, virtual adversarial training method is used to regularize the network, introduce virtual noise into the input layer, and consistently maintain the output of the encoder. The model parameters are adjusted by combining the classification loss and virtual adversarial loss to train the classifier. During this process, PSO algorithm is used to optimize the global features and parameters of the model, the optimized framework is used for the MI classification task.

The major contributions of our study are given as follows:

(1) We proposed a novel algorithm TransEEGNet to improve EEGNet with self-attention mechanism for MI-BCI classification task, through measuring the intensity of attention between different nodes, which can yield decoding accuracy and computing efficiency comparing with existing solutions.
(2) We applied hybrid particle swarm optimization in classification method, which allows optimization of the key parameters of extraction and classification process, in order to improve the classification efficiency of the optimized model.
(3) A VAT-EEGNet method was proposed to build smooth regularization constraints of TransEEGNet, which avoids the overfitting problem caused by the limited sample datasets, and improves the robustness of the model against random and local perturbations.
(4) We also evaluate our methods on a Competition dataset of BCI, and the results verify the effectiveness of our proposed approach.

The remainder of this paper is as follows. Related work is described in Section 2. Section 3 introduces the details on our framework. The practical results and discussion are presented in Section 4, and the conclusions and future work are discussed in Section 5.

## 2. Related Work

(1) Attention is a mechanism for organisms to flexibly allocate limited computing resources. Deep learning attention mechanism was first proposed to solve the problem of Natural Language processing (NLP). It behaved well in many NLP task fields, proving the great potential of the model structure. Transformer is also being studied in fields other than NLP. Carion et al. [38] combined CNN with transformer model and applied it to image detection and segmentation. This method first learns image features using CNN, then inputs the features into the encoding component, and obtained classification results and frame positions through decoding part. Dosovitskiy et al. [39] divided images into small areas of fixed size, and employed linear projection of these small areas by adding location coding. Transformer was subsequently used for classification, which could reach or partially exceed the best level of current image classification. This approach also reduced the amount of computation required. Srinivas et al. [40] replaced the spatial convolutions with self-attention module in the final three bottleneck blocks of a ResNet, achieving an excellent show of 84.7% accuracy on the ImageNet benchmark. Wang et al. [41] introduced the Pyramid Vision Transformer (PVT) as backbone network, which improved the performance of downstream tasks, surpassed other network structures in various dense prediction tasks, and reduced the computation time for large feature maps.

(2) The training is prone to overfitting when a sample size with labels is insufficient. Regularization is a method used to prevent overfitting in deep learning. Common regularization methods do not take into account input data distribution, such as L1 and L2 regularization and dropout. Miyato et al. [42] proposed local distributional smoothness (LDS) to promote the model distribution smoothness, which determined the adversarial direction from the model distribution alone without using the label information. Reference [43] proposed Virtual Adversarial Training (VAT), a new regularization method based on virtual adversarial loss, which achieves state-of-the-art performance on SVHN and CIFAR-10 datasets. In addition, the computational cost of VAT is relatively low. Therefore, the VAT method was creatively introduced into the BCI to alleviate the overfitting problem caused by the small amount of sample data.

(3) Kennedy et al. [44] proposed Particle Swarm Optimization (PSO), a random search algorithm for finding the global optimal solution, which has fast convergence speed and few parameters. Deng et al. [45] proposed a gate allocation method based on PSO to allocate the flights to different gates with resource optimization. Therefore, the ability of this model to optimize the classification of motor imagination EEG signals was further explored.

From above studies, we found that deep learning methods for EEG signal classification are usually based on convolutional neural networks, which results in unsatisfactory classification accuracy due to the lack of overall dependence of EEG signals in feature extraction. In addition, the existing methods usually use dropout and L2 regularization methods to solve the overfitting problem caused by small-scale datasets. However, the generalization capability needs further improving since these methods neglect the distribution of input data and optimization is not well performed [46].

## 3. Methods

### 3.1. Preprocessing

Data preprocessing can remove many artifacts from the collected signals, reduce noise interference, and improve the signal-to-noise ratio. However, different signal frequency ranges must be considered, and task-irrelevant components must be removed in different applications. The preprocessing of raw EEG data includes segmentation, band-pass filtering, and standardization. In this study, 2 s of data are used from 0.5 s after the prompt to 2.5 s after signal collection, and a total T of sampling points (T = 2 * 250) is used. The data length is N, and the dimensions of sample data are represented as the number of channels C * sampling points T. A 4–40 Hz band-pass filter is used to remove baseline drift and frequency noise while preserving α and β rhythms related to MI. A Z-score standardized method is used to alleviate the non-stationarity of the data, which is expressed as:

(1) $$X=\frac{x-\mu }{\delta }$$

where *X* represents standardized data, *x* represents input signal data, *μ* and *δ* represent mean and standard deviation of the training data, respectively.

### 3.2. TransEEGNet

We extend EEGNet by incorporating the attention information between any two nodes because the attention information can be crucial for understanding the global dependence of signals. We built a model combining EEGNet and transformer, the convolution layer was used to extract spatial and temporal information respectively, and then features through the attention module were further learned. The obtained features were connected and inputted into the full connection layer.

**Figure 1:**
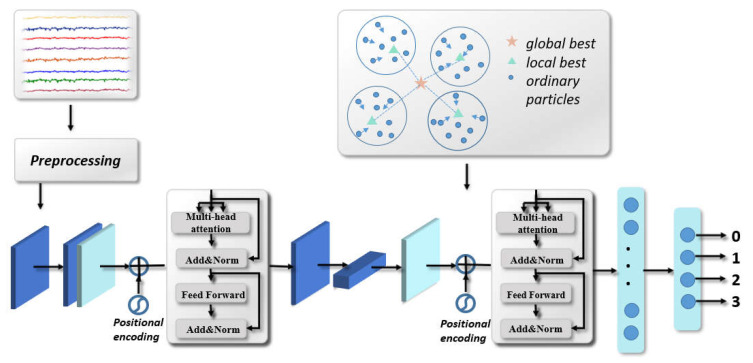
The overall framework of TransEEGNet method.

The TransEEGNet framework is illustrated in Figure 1. The first block (spatial convolution transformer) contains: (1) a convolutional layer (L1) uses 8 filters of size (1, 64) outputs feature maps containing the learned frequency band. Batch normalization (BN) is performed after the first convolutional layer [47]. The use of BN layer can help alleviate the ineffectiveness of weight initialization in the training process of the generator, which has good stability. This is closely followed by the depthwise convolutional layer (L2) with a kernel size of (22, 1), which is used to extract spatial information from the previous step. Subsequently, a batch normalization layer is applied and an average pooling layer with kernel size of (1, 4) is employed to downsample in the time dimension and integrate the information. Each convolution operation has a fixed receptive field, and the global feature information of the signal can only be obtained using multiple convolution operations. This leads to inefficient learning of the generated model and potentially missing critical information. The introduction of the self-attention mechanism can solve the size limitation of receptive field caused by convolution structure and enable characteristics to be used at all times to generate global information when generating feature vectors. The self-attention layer (L3) is used to directly calculate the similarity between nodes, and to weighted sum the features at all times, which obtain key information more effectively. Self-attention is expressed as follows:

(2) $$Attention\left(Q,K,V\right)=softmax\left(QK^{T}/\sqrt{d_{k}}\right)V$$

where *Q*, *K* and *V* are vector matrices created by multiplying the feature vectors by three corresponding weight matrices. The information of all the time points under each channel can be regarded as the characteristics of the channel. $QK^{T}$ represents the key vector (*K*), and the query vector (*Q*) is used to calculate the attention intensity between any two channels using dot product. The attention correlation matrix is then divided by a scaling factor of $\sqrt{d_{k}}$ to ensure the stability of gradient. The output attention score for each position was multiplied by each value vector (*V*). $Attention\left(Q,K,V\right)$ is the final weighted vector representation. Besides, a feed-forward neural network was employed to receive the previously obtained output vectors.

The second block contains several layers. (1) a depthwise convolutional layer (L4) with kernel size (1, 16) and a pointwise convolutional layer (L5) with kernel size (1, 1) which are used to extract the temporal information and connect multiple two-dimensional matrices. Next, a batch normalization layer is applied, followed by an average pooling layer with kernel size of (1, 4). The features obtained using only a set of weight matrices are relatively isolated. Therefore, a multi-head attention (MHA) [37] layer (L6) is employed to construct multiple groups of *Q*, *K*, and *V* in parallel, and subsequently execute attention operations in parallel. This allows the model to learn the dependencies from different angles through multiple attention modules. MHA is expressed as follows:

(3) $$MHA\left(X_{Q},X_{K},X_{V}\right)=\left[head_{0};\ldots ;head_{h-1}\right]\;W^{O}\;$$

(4) $$head_{i}=Attention\;\left(X_{Q}W_{i}^{Q},X_{K}W_{i}^{K},X_{V}W_{i}^{V}\right)\;\;\;$$

where $W_{i}^{Q}$, $W_{i}^{K}$, $W_{i}^{V}$ represent the weight matrices for each group of *Q*, *K* and *V*, respectively. $head_{i}$ represents the representation subspaces of ith attention head. MHA denotes splicing all groups of attention heads together and is multiplied by $W^{O}$ to obtain the final output matrix that contains all the attention head information. A feed-forward (FF) block (L7) contains two fully-connected layers, and the ReLU activation function is connected behind the multi-head self-attention layer to strengthen the expression and perception abilities of the model. Layer normalization is added after the multi-head self-attention layer to normalize the representation and improve the expression of the relevant component. The residual connection is used in the MHA and FF blocks to reduce model complexity.

### 3.3. Optimization

The PSO method was used to optimize the above model in order to achieve faster convergence speed and global optimum (reduce the effect of the local optimum). We developed an algorithm based on PSO, which is a population-based evolutionary computation method. The PSO approach is reviewed in this section.

The PSO algorithm first randomly initializes a bunch of particles, and then selects the optimal solution by gradually updating the flight speed and position at each iteration. This is accomplished by tracking the best and the global extremums found by the individual group, respectively. Vector ($x_{i1}x_{i2},\ldots ,x_{iD}$) is denoted as the position vector of particle i at time t, vector ($x_{i1}x_{i2},\ldots ,x_{iD}$) is denoted as the velocity vector of particle i at time t. In the optimization process, the velocity and position vectors of individuals in the next generation population are updated using the following:

(5) $$V_{ij}\left(t+1\right)=\omega V_{ij}\left(t\right)+c_{1}r_{1}\left(P_{ij}\left(t\right)-X_{ij}\left(t\right)\right)+c_{2}r_{2}\left(P_{gj}\left(t\right)-X_{ij}\left(t\right)\right)\;$$

(6) $$X_{ij}\left(t+1\right)=X_{ij}\left(t\right)+V_{ij}\left(t+1\right)$$

where i = 1,2,...,N, j = 1,2,...,D, N is the group size, w is the inertia weight, r1 and r2 are uniformly distributed random numbers in the interval (0,1), C1 and C2 are the acceleration coefficients. $P_{ij}$ represents the local best in the jth dimension, $P_{gj}$ represents the global best in the jth dimension. Lastly, t denotes the number of current iterations.

The hybrid PSO-Adam-EEGNet neural network training stage mainly includes PSO-Adam-EEGNet structure design, PSO global optimization, Adam local optimization, and other key steps. In the global optimization stage, PSO is used to optimize the initial training point to the vicinity of the global extremum point, so that the training can skip the local sub-optimal points and saddle points. This partially alleviates the problem of local sub-optimal convergence and saddle point residence in training. Adam is utilized for the local optimization stage, so that the training can adaptively calculate the learning rate for each parameter, reduce the impact of the selection of learning rate and other parameters on the performance of the algorithm, simplify the implementation of the algorithm, and improve performance stability.

Algorithm 1 summarizes the hybrid optimization approach. First, the particle population size N, particle search space D, and other algorithm parameters are initialized, and the flying speed and position of particles are randomly initialized. Second, the fitness for p is computed using the standard fitness evaluation critic. Then, p is compared and it is determined whether to update using fitness. The optimal solution is found from the individual historical optimal solutions of all the particles. Third, the positions and velocities of all particles are calculated and updated. All particles continue to iterate in the search space until a satisfactory solution is obtained or other termination conditions are reached (the number of iterations overflows or the fitness meets the requirements). Here, the minimum optimization function and corresponding weight value are obtained. Subsequently, the initial weights of Adam optimization strategy are assigned as the parameter weights obtained by optimizing convolution network with PSO to accelerate the convergence of the model and reach the optimal value.

Specifically, the inertia weight for the PSO parameter setting is assigned a value of 0.64. The acceleration coefficients c1 and c2 are set assigned as 1.524, the population size is set as 1932 (The parameters in transformer are fixed, and the other parameters are optimized) In addition, the number of iterations is set as 10. All the parameter values were finally selected after many empirical attempts. Finally, PSO optimizes the EEG parameters to obtain the optimal decoding model.
**Algorithm 1** Hybrid PSO-Adam-EEGNet method (PAE)**Require:** Population size $P_{N}$, generation $max_{gen}$σ, exponential decay rates for moment estimates,
$\rho_{1}$ and $\rho_{2}$ in [0,1), small constant δ used for numerical stabilization, **Input:** Initialize the particles randomly until the total number of particles reach $P_{N}$, $g_{best}$i ← Empty, 0 Initial parameters θ Initialize 1st and 2nd moment variables
s = 0, r = 0 Initialize time step t = 0 while stopping criterion not met, do   while $i\;\text{<}\;max_{gen}$ do  for particle p in $P=\left\{P_{1},P_{2},\ldots ,P_{N},\right\}$ do    p ← Position update of p using standard PSO operation.    fitness ← Compute the fitness for p through the TransEEGNet    Fitness update of p by fitness  if fitness > fitness of the personal best then    Update the personal best of p with the p;    end if  end for   $g_{best}$
$\text{←}\;\mathrm{Best}\;\mathrm{particle}\;\mathrm{updation}\;\mathrm{among}\;\mathrm{the}\;\mathrm{current}\;g_{best}$   i ← i + 1;  end while  $\mathrm{return}\;g_{best}$  Post process it by sending it to the TransEEGNet.  While iter
$\text{<}\;max_{iter}$ do    Sample a minibatch of m
$\mathrm{examples}\;\mathrm{from}\;\mathrm{the}\;\mathrm{training}\;\mathrm{set}\left\{x^{\left(1\right)},\ldots ,x^{\left(m\right)}\right\}$
$\mathrm{with}\;\mathrm{corresponding}\;\mathrm{targets}\;y^{\left(i\right)}$.    $\mathrm{Compute}\;\mathrm{gradient}\text{:}g\leftarrow \frac{1}{m}\nabla_{\theta }\sum_{i}L\left(f\left(x^{\left(i\right)};\theta \right),y^{\left(i\right)}\right)$                         t←t+1    $\mathrm{Update}\;\mathrm{biased}\;\mathrm{first}\;\mathrm{moment}\;\mathrm{estimate}\text{:}s\leftarrow \rho_{1}s+\left(1-\rho_{1}\right)g$    $\mathrm{Update}\;\mathrm{biased}\;\mathrm{second}\;\mathrm{moment}\;\mathrm{estimate}\text{:}\;r\leftarrow \rho_{2}r+\left(1-\rho_{2}\right)g⊙g$    $\mathrm{Correct}\;\mathrm{bias}\;\mathrm{in}\;\mathrm{first}\;\mathrm{moment}\text{:}\;\hat{s}\leftarrow \frac{s}{1-\rho_{1}^{t}}$    $\mathrm{Correct}\;\mathrm{bias}\;\mathrm{in}\;\sec\mathrm{ond}\;\mathrm{moment}\text{:}\;\hat{r}\leftarrow \frac{r}{1-\rho_{2}^{t}}$    $\mathrm{Compute}\;\mathrm{update}\text{:}∆\theta =-\sigma \frac{\hat{s}}{\sqrt{\hat{r}}+\delta }$ (operations applied element-wise)    Apply update:θ←θ+∆θ  end while  return θ


### 3.4. VAT-TransEEGNet

**Figure 2:**
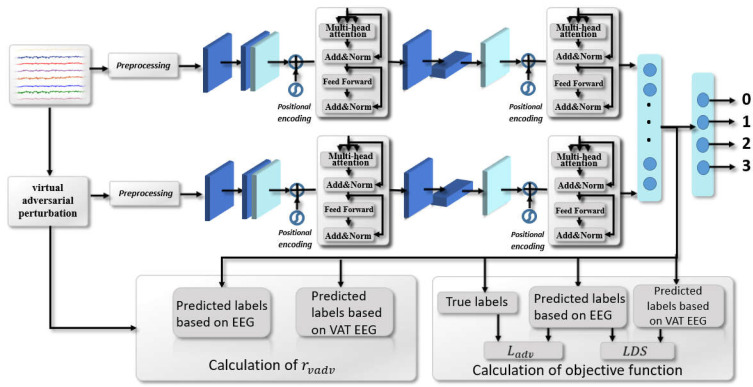
The flowchart of VAT-TransEEGNet model pipeline, with the objective function consisting of two parts.

The sample size of motor imagery EEG signal data is typically small, which possibly cause overfitting phenomenon. To solve this problem, we adopted the VAT strategy in the decoding process, and a VAT-TransEEGNet model was proposed to adjust network parameters together with the classification loss function and virtual adversarial loss. In addition, the classifier was trained to improve the decoding accuracy. The VAT strategy trains TransEEGNet model by adding virtual adversarial perturbation to improve its generalization performance. The schematic diagram of VAT-TransEEGNet model is shown in Figure 2.

First, the perturbated EEG signal series was obtained by combining the EEG signal series with the virtual adversarial perturbation $r_{vadv}$. Second, the TransEEGNet model with hybrid optimization approach was employed to extract the features of EEG signal series and the perturbated EEG signal series, respectively. Third, a fully connected layer was utilized to predict the labels of data samples based on the extracted features.

Formally, adversarial training is performed on marked data, and its loss function of adversarial training is expressed as:

(7) $$L_{adv}\left(x_{l},\theta \right):=D\left[q\left(y|x_{l}\right),p\left(y|x_{l}+r_{adv},\theta \right)\right]\;$$

(8) $$r_{adv}:=\underset{r;||r||\leq \epsilon }{\arg\;\max}D\left[q\left(y|x_{l}\right),p\left(y|x_{l}+r,\theta \right)\right]$$

where $D_{l}=\left\{x_{l}^{\left(n\right)},y_{l}^{\left(n\right)}|n=1,\ldots ,N_{l}\right\}$ denotes a labeled EEG signal dataset, $D_{ul}=\left\{x_{ul}^{\left(m\right)}|m=1,\ldots ,N_{ul}\right\}$ denotes an unlabeled dataset which combine with virtual adversarial perturbation. $q\left(y|x_{l}\right)$ represents the real label distribution of the training sample $x_{l}$, $p\left(y|x_{l},\theta \right)$ represents the predicted label distribution of $x_{l}$ when the model parameter is θ, and $D\left[q,p\right]$ is the Kullback-Leibler (KL) divergence, which is used to evaluate the distance between *P* and *Q*. $r_{adv}$ represents the disturbance vector that can maximize the prediction deviation of $x_{l}$, which is the disturbance direction. The adversarial loss $V_{loss,1}$ can be obtained by minimizing the KL divergence between the two outputs, which is expressed as:

(9) $$V_{loss1}\left(x_{l},\theta \right)=-L_{adv}\left(x_{l},\theta \right)$$

Virtual adversarial loss computes the adversarial direction based on the virtual labels, approximate replace unknown real labels with current model output. This is expressed as:

(10) $$DS\left(x_{*},\theta \right):=D\left[p\left(y|x_{*},\hat{\theta }\right),p\left(y|x_{*}+r_{vadv},\theta \right)\right]$$

(11) $$r_{vadv}:=\underset{r;{\left||r|\right|}_{2}\leq \epsilon }{\arg\;\max}D\left[q\left(y|x_{*},\hat{\theta }\right),p\left(y|x_{*}+r\right)\right]$$

where $x_{*}$ represents either $x_{l}$ or $x_{ul}$, $\hat{\theta }$ is the model parameter vector in a certain iteration during training, ϵ is the norm constraint for adversarial direction, LDS is a function of the local smoothness of the current model at each input data point *x*, $r_{vadv}$ is the virtual adversarial perturbation that can enhance the local smoothness of the model. Next, the weight is updated to minimize the KL divergence and the virtual adversarial loss is given by:

(12) $$V_{loss2}\left(x_{*},\theta \right)=\alpha \frac{1}{N_{l}+N_{ul}}\underset{x_{*}\in D_{l},D_{ul}}{\sum }LDS\left(x_{*},\theta \right)$$

where α is the regularization coefficient that controls the trade-off between the cross-entropy loss and the virtual adversarial training loss. The full objective function is thus given by:

(13) $$V_{loss}\left(x_{l},x_{*},\theta \right)=V_{loss1}\left(x_{l},\theta \right)+V_{loss2}\left(x_{*},\theta \right)\;\;$$

Softmax was employed as the classifier in the VAT-TransEEGNet model to construct supervised classification losses. The cross-entropy between the predicted value and the real label was calculated and the supervised classification loss was obtained, which is expressed as:

(14) $$C_{loss}=-\frac{1}{M}\overset{K}{\underset{k=1}{\sum }}\overset{N}{\underset{n=1}{\sum }}y_{n}^{k}\log\left({\hat{y}}_{n}^{k}\;\right)$$

where *N* is the number of trials and *K* is the number of categories. $y_{n}^{k}$ denotes the corresponding target value, and ${\hat{y}}_{n}^{k}$ represents the predicted probability of the *n*-th trial for the category k. According to Equations (13) and (14), the total loss function of the training classifier can be expressed as:

(15) $$L_{loss}=V_{loss}+C_{loss}\;$$

The virtual adversarial loss function with the supervised loss function were combined and the network parameters were jointly adjusted. This improved the robustness of the model to virtual adversarial perturbations by minimizing the loss function. Then, the generalization of the model was further improved through VAT training.

## 4. Experiments and Results

### 4.1. A. Study Design

#### 4.1.1. Dataset

This study pertains to BCI Competition IV IIa dataset [48], a public dataset including the motor imagery records of nine subjects recorded at a signal sampling rate of 250 Hz and 22 electrode channels. The dataset is openly available at https://bbci.de/competition/iv/ (accessed on 10 October 2021). Subjects completed four types of motor imagination tasks (left hand, right hand, feet and tongue). For each subject, two groups of tasks were recorded on different two days. Each group of tasks included six sections, with a short rest in the middle. Each section included 48 single experiment trials, and each group of tasks had 288 trials in total. The original EEG signal was filtered by a band pass filter of 0.5–100 Hz. The MI phase extracted in each experiment adopts the same time window, the input signals of the experiments consisted of a time series of 22 channels with 500 sampling points (22 × 500). The dataset was divided into training set and test set. The data from one subject were used as a test set, and the data from the other eight subjects served as training set.

**Figure 3:**
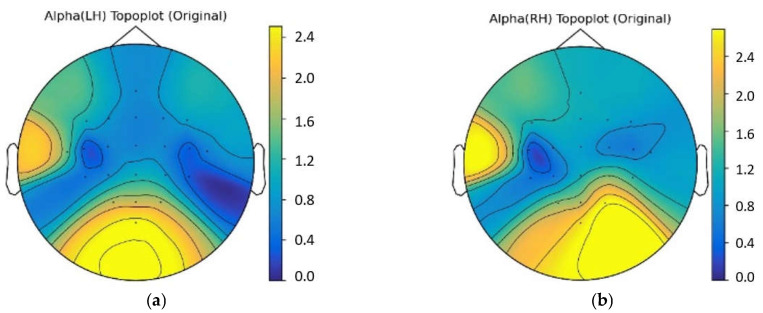
The topographical distribution of left-hand and right-hand data for a typical subject in alpha band. (**a**) The topographical map of left-hand data; (**b**) The topographical map of right-hand data.

In this work, the dataset we used is an open available dataset instead of our own one, and it is one of the most frequently employed open dataset in MI decoding. In other MI decoding studies used the same dataset, the spatial distribution of power changes of multi-channel EEG in alpha band was depicted by topographical map [49]. Therefore, we also plotted the topographical map of data in the BCIC IV IIa dataset to show the spatial distribution of EEG. For instance, the topographical distribution of left-hand and right-hand data of a typical subject were shown in Figure 3. It can be seen that the deep blue region on the right side in Figure 3a reveals the ERD phenomenon related to left hand motor imagery, and the deep blue region on the left side in Figure 3b reveals the ERD phenomenon related to right hand motor imagery.

#### 4.1.2. Model Parameter

The proposed method was implemented with Python 3.7 and the PyTorch library on a GeForce 3090Ti GPU. Scikit-learn was employed to calculate the confusion matrix.

The batch size in the experiment was set as 64, the number of attention heads in each layer was set to 8, the dropout was set to 0.3, the parameter of the feedforward neural network part was set to 512, and the weight attenuation was 0.0001. The inter-subject models were trained for integrity. Wilcoxon Signed-Rank Test was utilized to analyze the statistical significance.

#### 4.1.3. Compared Methods

We compared five representative methods published in recent years. Details of method and implement are given as follows.

- FBCSP [12]: FBCSP was used as the baseline method. CSP was used for feature extraction with one-vs-rest (OVR) strategy, and the support vector machine was used for inter-subject classification.
- DeepConvNet [24]: DeepConvNet is a general-purpose architecture that consists of five convolutional layers. We trained this model in the same way we train the TransEEGNet model.
- EEGNet [22]: EEGNet designs a lightweight CNN for EEG decoding. As its method was designed for 128 Hz EEG signals (as opposed to 250 Hz signals used in this study), adjustments were performed according to Borra et al. [50].
- ShallowConvNet [24]: ShallowConvNet was designed as a lightweight CNN with a temporal and spatial convolutional layer. Our implementation was performed according to Schirrmeister et al.
- MCNN [51]: MCNN uses a multilayer perceptron and autoencoders for fusing the CNN model to improve EEG decoding performance. The experiment parameters were adjusted according to the origin study.

### 4.2. Experiment Results

#### 4.2.1. Comparison Experiments

We compared the performance of our model with the performance of other typical deep learning-based methods on the EEG dataset. The inter-subject experiments were conducted with nine subjects for all comparison experiments. The results of the performance comparisons and accuracy of each classification method for pattern classification with 2 s of data are listed in Table 1. The VAT-TransEEGNet model reaching an average classification accuracy of 63.56%, which exceeded that of the fiducial CNN model and reaching the best level in all the other optimized models. For FBCSP, which is a classic machine learning method, distinguishing features extracted from one subject to another was poor, and individual differences were ignored. ShallowConvNet and DeepConvNet methods represent two different architectures proposed in the same paper that directly use the spatio-temporal two-dimensional matrix of EEG signal without fully considering the topological relationship between EEG electrodes. Therefore, the average cross-subject classifications of the ShallowConvNet and DeepConvNet methods were 16.26% and 24.15% lower than the proposed model, respectively. The deep learning multi-layer CNN (MCNN) [50] fuses CNNs with different architectures. It only achieved a 55.39% average classification accuracy in the cross-subject classification on BCIC IV-2a dataset, which was 8.17% lower than our proposed method.

**Figure 4:**
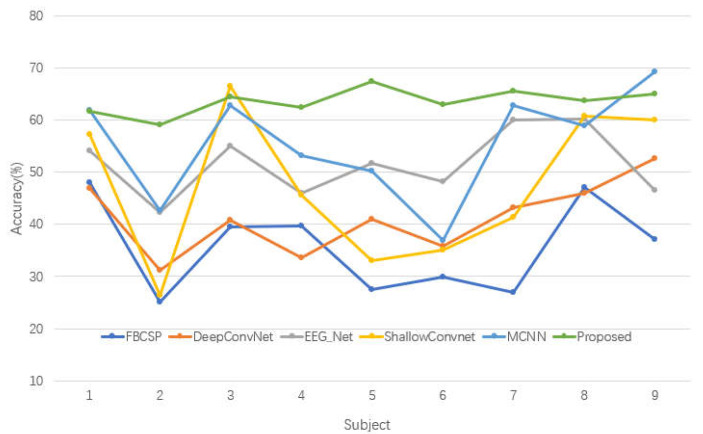
Visual diagram of comparative experiment.

The accuracies of proposed method on subjects 2, 4, 5, 6, 7, 8 were higher than those of the comparison methods, as shown in Figure 4. Additionally, the proposed model achieved relatively better average classification accuracy compared with other models. The outcome indicates that our proposed VAT-TransEEGNet model outperformed the compared methods significantly (*p* < 0.05). Furthermore, the standard deviation (std) of our method is 11.54 which is lower than DeepConvNet, EEGNet, ShallowConvnet. By comparison, the std of proposed method was moderate, which demonstrates that the stability of our proposed method is acceptable.

#### 4.2.2. Ablation Study

An ablation study was conducted to verify the necessity of each component in VAT-TransEEGNet model, and the result is shown in Figure 4.

**Figure 5:**
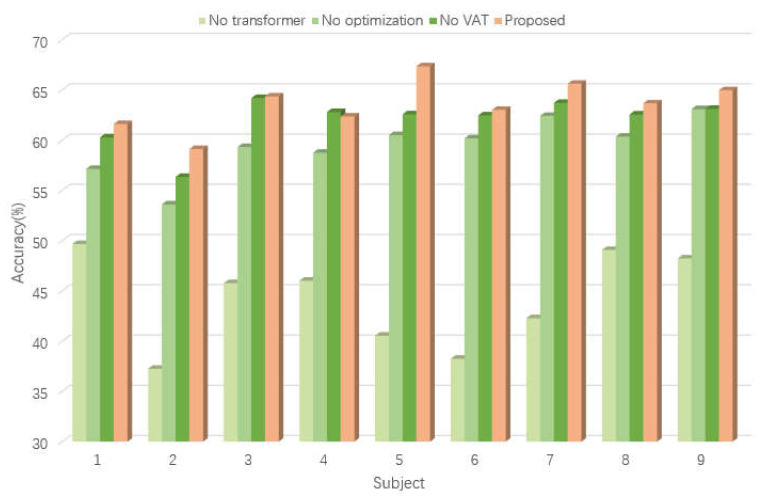
Visual diagram of ablation study.

(a) we removed the transformer block from the VAT-TransEEGNet module;
(b) we removed the PSO-Adam-EEGNet method from the VAT-TransEEGNet module;
(c) we removed the VAT process from the VAT-TransEEGNet module;

Figure 5 shows the result that the accuracy decreased significantly (19.43%) in case a, which indicates that the transformer mechanism played the most important role. Additionally, the mean accuracy decreased by 4.07% for case b, which indicates that the PSO-Adam-EEGNet method had a meaningful effect on decoding performance. The accuracy for case c decreased from 63.56% to 62.01% without the VAT block. Furthermore, VAT demonstrated a distinct improvement of 4.77% and 1.85% for subjects 5 and 9, respectively. Figure 5 intuitively shows that the fusion model performed better than the single block model with a substantial improvement in accuracy. This illustrates that the proposed model is reasonable and robust in cross-subject classification.

#### 4.2.3. Algorithm Performance

The proposed VAT-TransEEGNet model was initially evaluated on the public dataset to solve the MI classification task. The performance of the algorithm was experimentally tested as follows.

We tested the category-based model performance on the basic of (a) confusion matrix, (b) accuracy, precision, recall, F1-score and specificity, (c) different number of attention heads.

**Figure 6:**
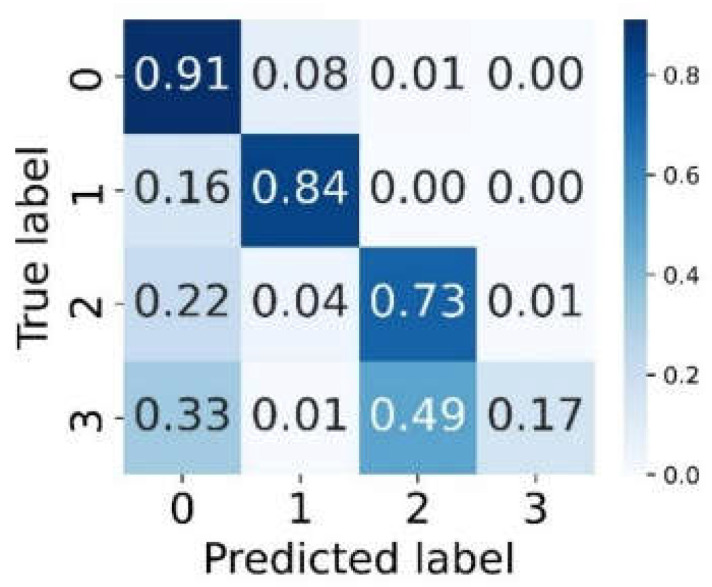
Visualization of confusion matrix.

(a) The confusion matrix points the specific numbers for each classification. The classification task has a total of four categories in this study. The confusion matrix is 4 × 4, where 0 represents the left-hand motor imagination category, 1 represents the right-hand motor imagination category, 2 represents the both feet motor imagination category, 3 represents the tongue motor imagination category. It can be seen that the classification accuracies of left and right hand are higher than those of the other two categories in the four categories, as shown in the visualization of confusion matrix in Figure 6. Misclassification mainly occurred when tongue imaginary movements are classified as foot imaginary movements or left-hand imaginary movements.

This indicates that the model has learned some sample features and could distinguish categories with large differences between classes, such as left-hand and right-hand imaginary movements. However, tongue and feet categories with small differences between classes were easily confused because of the complexity of thinking activities between subjects.

In general, our model has a better classification effect for cross-subjects since it learns global features and gets more abundant features with a high contribution to the classification via the self-attention mechanism to obtain the correlation between electrodes and the sampling points.

(b) On this basis, the performance on five basic evaluation indicators was further studied on the proposed model: accuracy, precision, recall, F1-score, and specificity for each class. The results are displayed in Table 2.

The accuracy rate is the proportion of all samples in which the category of the sample is correctly classified. Recall indicates the proportion of positive cases in the sample which is correctly predicted. The recalls of left and right hands were 90.97% and 84.03%, respectively, which were higher than those for both feet and tongue. In addition, the average accuracy of right-hand motor imagination was highest in the four class tasks, with a value of 92.71%.

(c) We also tested the performance with different attention head number settings on model. Table 3 shows the average accuracy with numbers of attention heads set to 1, 4, 6, 8,16. The classification accuracy improved when the number of attention heads increased. The highest accuracy of 63.56% was achieved when the number of attention heads is 8. The accuracy rate remained basically unchanged, with a slight decrease after exceeding eight layers.

#### 4.2.4. Interpretability and Visualization

The distribution of classification results for real data samples is visualized using the t-distributed stochastic neighbor embedding (t-SNE) method [52].

**Figure 7:**
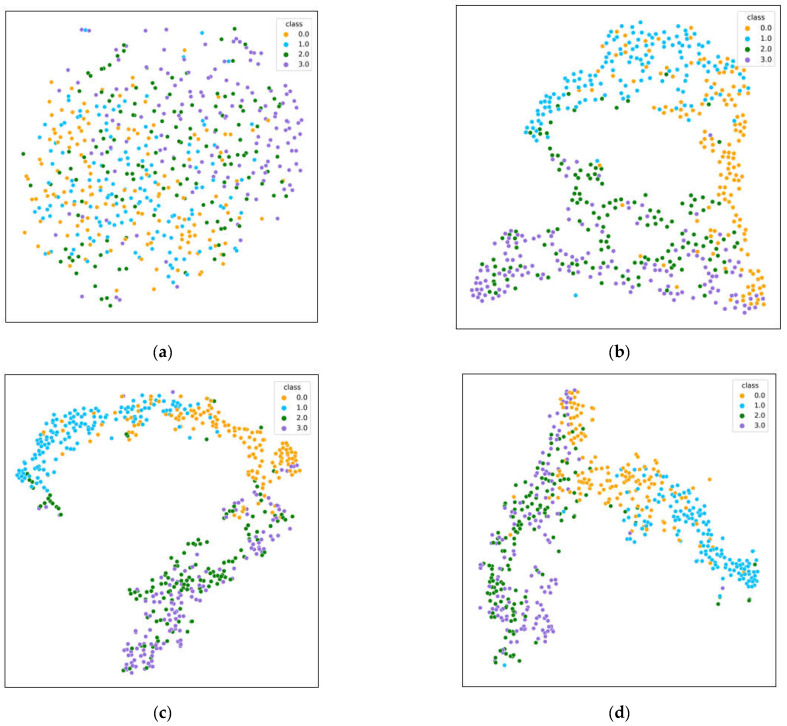
Visualization with t-SNE. (**a**) Data distribution of initial data. (**b**) Data distribution after the transformer block. (**c**) Data distribution after the optimization block. (**d**) Data distribution after the VAT block, also the input of the classifier.

A visual display of the products is shown in Figure 7. Yellow, blue, green, and purple represent the left hand, right hand, foot and tongue motor-imagination EEG signals, respectively. The best low-dimensional simulated data points were obtained by minimizing the KL divergence. The quality of the classification model was analyzed from the perspective of visualization.

The initial data distribution and data distribution after the implementation of transformer block are shown in Figure 7a,b, respectively. Each category of EEG signals shows clustering distribution characteristics in two-dimensional space. The data distribution after the hybrid optimization block with some intersecting areas for the four types is shown in Figure 7c. The data distribution before classification indicates that the four categories were relatively distinguishable, as shown in Figure 7d.

Each category of EEG signals shows obvious clustering distribution characteristics in two-dimensional space, as shown in Figure 7a–d. Specifically, the left hand and right hand categories were obviously distinguished, the other two categories had a certain degree of overlap. Therefore, the proposed method exhibits a relatively good distinguishing ability for the four categories.

## 5. Discussion

### 5.1. Framework Analysis

In this study, the proposed VAT-TransEEGNet model improved the accuracy by fusing the global dependence and global optimization of EEG signal sequence.

CNN is often used for feature extraction and classification in BCI [18]. However, it cannot obtain the correlation between long-sequence nodes. Transformer model is commonly used in the field of natural language processing, in which the attention structure can calculate the attention weights between any two nodes. But attention block exhibits a relatively weak ability to extract detailed local information. Therefore, we combine the advantages of Transformer to obtain global dependencies of EEG signals and the strong feature extraction ability of CNN. The VAT-TransEEGNet structure was proposed to improve the performance of motor imagery tasks. According to Table 1, our proposed structure did not achieve the best accuracy on subject 1, subject 3 and subject 9. However, it did significantly improve the accuracy for the other six subjects. The proposed method achieved optimal classification performance across subjects compared to the other representative algorithms (FBCSP, improved convnet, etc.). Therefore, this verifies the strong competitiveness of using the attention mechanism to process EEG characteristics and optimize the training process in BCI.

The influence of the corresponding meliorative features on classification results is shown in Figure 5. The accuracy of the model was lowest when the proposed method did not use a self-attention mechanism. This is because the addition of the self-attention mechanism focused more attention on valuable feature information for classification results by calculating the correlation of features in long sequences. This enabled the network to obtain more abundant and distinguishable features. The PSO algorithm was applied to the proposed framework and combined with Adam algorithm to optimize the model presented in Ref. [44]. The accuracy decreased without optimization methods, indicating the effectiveness of the addition of hybrid optimization algorithms, as shown in the ablation study in Figure 5.

We further analyzed the classification of each category and the misclassification of each category through the confusion matrix. The number on the diagonal of the confusion matrix represents the number of correct classifications of the category. According to Figure 6, the left-hand and right-hand motor imagination can be well distinguished in the four class tasks. The recalls for the both feet and tongue were lower than those of left-hand and right-hand motor imaginations, which may be due to the fact that each subject had their own diversity, causing the physiological signals to be more obvious during the left-hand or right-hand movement imaginations. Different from most previous studies based on intra-subject tasks, this study focused on inter-subject tasks, which have better generalization and practical significance for real-world EEG signal decoding tasks.

### 5.2. The Influence of Different Number of Attention Heads on Model Results

The Transformer model performs better with the addition of attention modules in NLP [36]. The classification accuracy continuously improved as the number of attentional heads increased to eight, as shown in Table 3. This is because each attention head focused on a different location to avoid extracting single features. Increasing the number of attention heads assisted in learning more comprehensive features, preventing the model from relying on certain features for classification, which leads to poor robustness. Accuracy decreased after exceeding eight layers due to the addition of attention layers may cause excessive parameters and led to model overfitting while the EEG signal dataset is generally insufficient. Therefore, there would be a better performance and less computation when eight attention heads were selected.

### 5.3. The Influence of Hyper-Parameter of VAT-EEGNet

**Figure 8:**
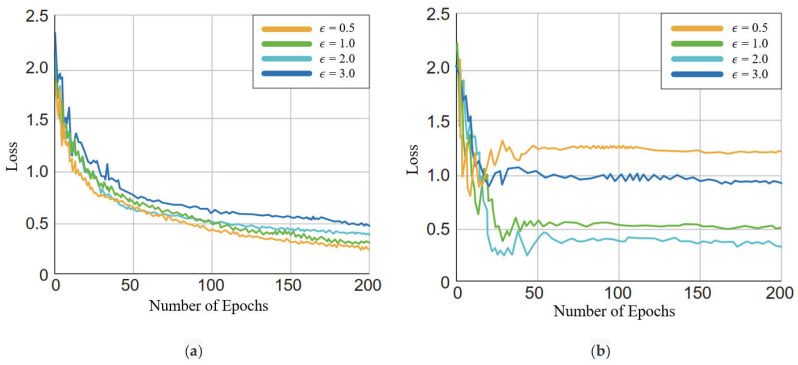
The loss function curves with different values of ϵ. (**a**) The loss function curves of training dataset. (**b**) The loss function curves of testing dataset.

In order to explore the impact of ϵ on model performance, we compared the loss values of four-class classification tasks with different ϵ values on the EEG dataset, and conducted comparative experimental analysis on training set and testing set when the model tended to converge in Figure 8.

Perturbation size ϵ is the hyperparameter of the VAT-EEGNet model. From Figure 8a, it can be observed that the training set with a larger value of ϵ had a higher loss value. The loss value of the testing set tended to be stable during the training process, with the lowest stable value at ϵ = 2.0, as shown in Figure 8b. Tuning the perturbation size ϵ was adequate for achieving suitable performance, which was validated in the study [43]. The results showed that the proposed method enhanced the robustness of model distribution against random perturbations, combined with the adjustment of the perturbation size.

Although the proposed method has achieved good results, the number of parameters still needs further reducing to avoid overfitting problem, which can improve the generalization capability on other datasets. Therefore, our future work includes (1) improving the framework to facilitate its application in motor imagery tasks of joint by parameters tuning and structure optimization, and (2) exploring ways to reduce the attention module parameters to increase model efficiency of Transformer model.

## 6. Conclusions

In this study, a novel feature selection and optimization method was explored for the inter-subject MI task. We proposed an innovative framework relied on self-attention mechanism to perceive global features of EEG signals. Considering that the limited size of datasets can cause overfitting problem, a VAT-TransEEGNet method was proposed that worked together with the self-attention mechanism. The experimental results showed that the VAT-TransEEGNet achieved noticeable classification performance and improved the anti-noise generalization ability in multi-classification EEG tasks.

## Figures and Tables

**Table 1:** Comparison of experimental results (the highest accuracy is bolded).

Method	FBCSP (%)	DeepConvNet (%)	EEGNet (%)	ShallowConvNet (%)	MCNN (%)	Proposed (%)
Subject 1	47.95	46.88	54.06	57.26	61.84	61.61
Subject 2	25.03	31.14	42.34	26.32	42.60	**59.12**
Subject 3	39.44	40.76	55.02	66.45	62.75	64.35
Subject 4	39.73	33.54	45.88	45.60	53.22	**62.36**
Subject 5	27.56	41.02	51.70	33.02	50.15	**67.35**
Subject 6	29.86	35.81	48.12	34.97	36.98	**63.02**
Subject 7	26.97	43.12	59.95	41.26	62.80	**65.62**
Subject 8	47.15	45.97	60.26	60.78	58.92	**63.67**
Subject 9	37.12	52.65	46.55	60.05	69.26	64.97
Average	35.65	41.21	51.54	47.30	55.39	**63.56**
Std	10.04	13.26	12.24	12.82	11.27	11.54

**Table 2:** Scoring performance.

Category	Accuracy (%)	Precision (%)	Recall (%)	F1-Score (%)	Specificity (%)
0	79.86	55.98	90.97	69.31	76.16
1	92.71	86.43	84.03	85.21	95.60
2	80.72	59.32	72.92	65.42	83.33
3	78.99	86.96	16.67	28.40	97.31

**Table 3:** Accuracy with different attention heads.

Numbers of Heads	Average Accuracy(%)
1	62.65
4	62.97
6	63.48
8	63.56
16	62.75

## Data Availability

The data presented in this study are openly available at the following URL/DOI: https://bbci.de/competition/iv/ accessed on 10 May 2022.
